# The Role of CD44 in Disease Pathophysiology and Targeted Treatment

**DOI:** 10.3389/fimmu.2015.00182

**Published:** 2015-04-21

**Authors:** Andre R. Jordan, Ronny R. Racine, Martin J. P. Hennig, Vinata B. Lokeshwar

**Affiliations:** ^1^Sheila and David Fuente Program in Cancer Biology, University of Miami-Miller School of Medicine, Miami, FL, USA; ^2^Department of Urology, University of Miami-Miller School of Medicine, Miami, FL, USA; ^3^Department of Urology and Uro-oncology, Hannover Medical School, Hannover, Germany; ^4^Department of Cell Biology, University of Miami-Miller School of Medicine, Miami, FL, USA; ^5^Miami Clinical Translational Institute, University of Miami-Miller School of Medicine, Miami, FL, USA

**Keywords:** CD44, hyaluronic acid, hyaluronidase, CD44-signaling

## Abstract

The cell-surface glycoprotein CD44 is involved in a multitude of important physiological functions including cell proliferation, adhesion, migration, hematopoiesis, and lymphocyte activation. The diverse physiological activity of CD44 is manifested in the pathology of a number of diseases including cancer, arthritis, bacterial and viral infections, interstitial lung disease, vascular disease, and wound healing. This diversity in biological activity is conferred by both a variety of distinct CD44 isoforms generated through complex alternative splicing, posttranslational modifications (e.g., N- and O-glycosylation), interactions with a number of different ligands, and the abundance and spatial distribution of CD44 on the cell surface. The extracellular matrix glycosaminoglycan hyaluronic acid (HA) is the principle ligand of CD44. This review focuses both CD44-hyaluronan dependent and independent CD44 signaling and the role of CD44–HA interaction in various pathophysiologies. The review also discusses recent advances in novel treatment strategies that exploit the CD44–HA interaction either for direct targeting or for drug delivery.

## Introduction

CD44 is a glycoprotein that is widely expressed on the surface of many mammalian cells, which includes endothelial cells, epithelial cells, fibroblasts, keratinocytes, and leukocytes (1). Extensive alternative splicing of nine variable exons and different combinational insertions results in many distinct CD44 splice variants. CD44 standard is the shortest and most abundantly expressed isoform; the other variants are expressed in a cell-specific manner and in the pathophysiology of many diseases. CD44 has several important physiological functions in cell–cell and cell–matrix interactions including proliferation, adhesion, migration, hematopoiesis, and lymphocyte activation, homing, and extravasation (2). This diversity in cellular activity is a result of variable expression of CD44 variants, post-translation modifications such as N- and O-glycosylation, and binding by a variety of ligands (2). The diversity of isoform expression, posttranslational modifications, the abundance and spatial distribution of CD44 on the cell surface are likely to be important for the regulation of signaling; in particular, because high molecular weight hyaluronic acid (HA) can bind multivalently to CD44 (3–12).

The non-sulfated glycosaminoglycan HA is the principle ligand of CD44. HA is a polymer of repeating disaccharide units d-glucuronic acid and *N*-acetyl-d-glucosamine and can range in size from millions of Daltons to small oligosaccharides. The size of the HA is important for its physiological functions. HA of a high molecular mass (HMM) can naturally bind multivalently to more surface receptors across a larger area of the cell than low molecular mass (LMM) HA, and this difference in the number of bound HA receptors allows different sizes of HA to have different signaling effects. HMM HA is typically >500 kDa, and LMM HA is typically between 10 and 500 kDa. Because of the lack of a standard size for HMM and LMM HA, this review will list the molecular mass of the HA fragments and will refer to the size as HMM or LMM based on the classification used by study authors. HA is a major component of tissue matrices and fluids and is involved in a variety of physiological functions such as maintaining tissue hydration and osmotic balance, cell proliferation, adhesion, and migration (13). Much of its regulation of cellular function is mediated through CD44 and RHAMM signaling, although it has been shown to affect toll-like receptor signaling as well. Since HA also plays a role in the assembly and remodeling of extracellular matrix, these activities of HA are also likely to affect cellular function. HA is synthesized by HA-synthases HAS1, HAS2, and HAS3 in humans. HA synthesis occurs on the cytoplasmic side of the plasma membrane and it is then expelled into the extracellular space. HA is synthesized by many cells but mesenchymal cells are the major source of HA. HA is degraded by hyaluronidase family of enzymes, and the well-studied hyaluronidases are HYAL1, HYAL2, and PH-20/SPAM1 (13). Hyaluronidases degrade HA by hydrolyzing the linkages between the d-glucuronic acid and *N*-acetyl-d-glucosamine disaccharides.

CD44 has been implicated in a number of diseases such as cancer, arthritis, interstitial lung disease (ILD), vascular disease, wound healing, and infections by pathogens. CD44–HA signaling has been found be involved in the pathophysiologies of both malignant and non-malignant diseases. While several excellent reviews have been focused on CD44–HA signaling and its implications in cancer (3–12), this review focuses on both HA-independent (mainly) and HA-dependent functions of CD44 in the pathophysiology of various diseases. In addition, the review discusses various strategies used for targeting of CD44, and CD44/HA-based therapeutic interventions and devices for targeted imaging and treatment options.

## CD44–HA Signaling and Human Disease

### Infections

The importance of CD44–HA signaling has been implicated in a number of studies. CD44–HA signaling plays important roles in host defense against invading pathogens, specifically in activation and migration of lymphocytes (14). HA signaling through CD44 has also been linked to generation of antimicrobial peptides (15). Several other studies have shown that CD44–HA signaling can be utilized by pathogens for progression of infections and resulting complications.

CD44–HA signaling has long been known to play a role in host defense through activation, homing, rolling, and extravasation of lymphocytes into inflammatory sites (16, 17). A recent study suggests that CD44–HA signaling also contributes to host defense during the early stages of infancy. CD44–HA signaling induces human β-defensin 2 (HβD2), which, in turn, enhances antimicrobial defense in the intestinal epithelium (15). The study found that HA derived from breast milk (milk-HA), at 2-week intervals up to 6-month postpartum when HA concentrations were highest, induced production of HβD2 in HT-29 cells, and mice fed with milk-HA also increased expression of MuβD3, the mouse ortholog of HβD2. The authors also fed CD44^−/−^, TLR4^−/−^, or wild type mice with milk-HA and found that MuβD3 expression was greatly reduced in CD44^−/−^ or TLR4^−/−^ mice when compared to wild type mice; HT-29 cells pretreated with anti-CD44 antibodies also showed inhibition of HβD2 expression upon treatment with milk-HA. *Salmonella* infection was shown to be greatly decreased in cells treated with milk-HA when compared to milk-HA pretreated with hyaluronidase. These results suggest that CD44–HA signaling is important in the establishment of intestinal epithelium resistance to invading pathogens during early infancy. In a previous study by the above-mentioned group, addition of LMM HA averaging 35 kDa was shown to upregulate HβD2 in HT-29 cells and in mice in a size-specific manner; similar expression was observed upon treatment in combination with HMM HA (2 MDa), but not with HA-2M alone (18). Interestingly, HA-35 up-regulation of HβD2 was shown to be toll-like receptor 4 (TLR4) dependent but not CD44 dependent. In the 2013 study, HβD2 up-regulation by milk-HA was both TLR4 and CD44 dependent. The 2013 study did not address whether breast milk HA of different sizes had any effect on HβD2 levels, and in the 2011 study CD44 dependency was not ascertained with HA-35 and HA-2M combination treatment. Other studies have also reported up-regulation of HβD2 and other antimicrobial peptides upon treatment with LMM HA (<200 kDa) in a CD44-independent manner (19, 20). TLR4 has been shown to complex with CD44 upon treatment with HA (21). Perhaps these studies together point to independent yet complementary mechanisms by which LMM HA (2.5 kDa) signals through TLR4, which may complex with CD44 in the presence of HMM HA (10 MDa), which has been shown to increase CD44 clustering (22).

### Bacterial infection

Group A *Streptococcus* (GAS) utilizes an intriguing method to escape host defenses and adhere to mammalian cells. The capsular polysaccharide of GAS comprises HMM HA that is similar in size to the HA synthesized by mammalian cells and tissues (23). It has been shown that GAS adheres to human keratinocytes through the binding of capsular HA polysaccharides to CD44 (24). An *in vivo* study by the same laboratory evaluated the importance of CD44 expression for GAS infection of the pharynx using C57BL/6 mice and K5-CD44 transgenic mice that expressed a CD44-antisense transgene (25). In this study, transgenic mice with reduced CD44 expression showed significantly lower GAS infection than mice with wild type CD44 expression. GAS infection was also reduced by treatment with anti-CD44 antibodies and addition of exogenous HA. This study further reinforced the idea that CD44–HA binding is important for GAS infection.

A more recent study evaluated the importance of the molecular mass of HA for macrophage-mediated phagocytosis of GAS in both *in vitro* and *in vivo* murine models (26). In this study, ingestion of GAS by macrophages was inhibited by addition of HMM HA (i.e., 800–1200 kDa), while the addition of LMM HA (i.e., 25–75 kDa) increased GAS internalization. Similarly, GAS survival was increased in murine blood in the presence of HMM HA. Interestingly, the study showed that treatment with hyaluronidase, an enzyme that degrades HA in small fragments increased internalization of GAS by macrophages. The internalization of GAS by macrophages was not present in transgenic mice expressing a CD44-antisense transgene even in the presence of LMM HA, demonstrating that CD44 expression on macrophages is required for GAS internalization. The HMM HA in the capsular polysaccharide of GAS mimics tissue homeostasis and allows GAS to escape detection by the host immune system. Contrarily, LMM HA (>200 kDa) may function as an endogenous danger signal, activating the innate immune system (27). CD44 has also been shown to function as a primary phagocytic receptor via HA signaling (28). Taken together, LMM HA may mediate a signaling cascade that leads to macrophage recruitment and facilitate phagocytosis of GAS by binding to CD44 expressed on macrophages. These studies suggest that CD44–HA signaling is important for GAS infection. Whether CD44–HA signaling aids in host defense of GAS infection depends on the molecular mass of HA, further exemplifying the intricacy of CD44–HA interaction and signaling in disease pathophysiology.

*Helicobacter pylori* infection is the major risk factor for gastric cancer, which is the second leading cause of cancer-related death in the world (29). Chronic infection by *H. pylori* has been shown to result in atrophy of acid-secreting parietal cells, which leads to increased proliferation of stem/progenitor cells in the isthmus (30). The increased proliferation of isthmus stem cells is believed to be one of the contributing factors that lead to neoplasia. A recent study showed that proliferation of isthmus stem cells after atrophy of parietal cells is a result of a signaling cascade that involves CD44–HA-mediated activation of ERK and STAT3 (31). The study showed that in CD44 knockout mice, or mice treated with PEP-1, an inhibitor of CD44–HA interaction, there are significantly less proliferating isthmus stem cells than in wild type mice after infection with *H. pylori*. CD44–HA signaling was also shown to be important in the progression toward gastric cancer after a complication of *H. pylori* infection. It would be interesting to see the impact of HA size in isthmus stem/progenitor cell proliferation after parietal cell atrophy. Parietal cell atrophy possibly leads to breakdown of HA to small fragments, which then induce to pro-proliferative signaling cascades; in that case, addition of HMM HA may serve to restore homeostasis and inhibit proliferation.

Pneumonia is caused by infection of lung parenchyma by numerous bacteria and is a leading cause of morbidity and mortality. A number of studies have implicated CD44 in the progression of bacterial infection and in the amelioration of lung inflammation in pneumonia models. The role of CD44 in the acute phase (6 h after infection) of pneumonia caused by *Escherichia coli* and *Streptococcus pneumoniae* differed significantly as reported in a previous study (32). The study showed that when compared to wild type mice, *E. coli*-induced pneumonia in CD44-deficient mice causes increased lung inflammation, as evidenced by increased neutrophil accumulation, migration, and increased mRNA levels of inflammatory genes. However, such was not the case in *S. pneumoniae*-induced pneumonia. The differences in *E. coli* versus *S. pneumoniae*-induced pneumonia may be because the latter expresses hyaluronidase, which breaks down HA. This would decrease CD44–HA-mediated signaling and the downstream induction of inflammatory pathways. The study found that HA levels were decreased in the lungs after Streptococcal infection. A lower dose of infection with *S. pneumoniae* may have given a clearer picture of CD44’s role in the inflammatory response to infection by this bacterium. A later study explored this scenario and found transient increases in inflammation in CD44 knockout mice at lower doses of *S. pneumoniae*-induced pneumonia (33). This study also looked at the role of CD44 in prolonged lung infection (10 days) by *Streptococcus*. The study found that CD44 knockout mice had less bacterial outgrowth and dissemination, and higher survival rates compared to wild type mice at lethal doses. Another study by the same laboratory found similar results for *Klebsiella pneumoniae*-induced pneumonia (34). Together these studies show that CD44 signaling is important for reducing inflammation in the lungs, and may increase bacterial dissemination and outgrowth. It is possible that CD44 anti-inflammatory signaling plausibly decreases the clearing of bacteria, and therefore, allows for their growth and dissemination to other sites. To this effect, CD44 has been previously shown to play an important role of resolving lung inflammation (35).

### Viral infection

CD44–HA signaling has also been shown to play an important role in the course of HIV and hepatitis C (HCV) infections. HIV virions have been shown to acquire functional CD44 from host cells (36–38). In a recent study, CD44–HA signaling was shown to affect the infectivity of HIV in unstimulated primary peripheral blood mononuclear cells, unstimulated CD4^+^ T cells, and M7-Lue cells (39). In this study, HIV with virions expressing CD44 showed decreased infectivity of unstimulated peripheral blood mononuclear cells, unstimulated CD4^+^ T cells, and M7-Lue cells when treated with exogenous HA. Interestingly, this decrease of infectivity was not seen in Jurkat-E6.1 cells, which do not express CD44 nor was this decrease in infectivity observed using HIV with virions that did not express CD44. The study also showed that HIV infectivity was increased upon addition of hyaluronidase, suggesting that endogenous HA plays a protective role against HIV infection. Cells treated with hyaluronidase had decreased HA at the cell surface and were five times more susceptible to HIV infection than cells not treated with hyaluronidase. The study also showed that exogenous HA reduces HIV infectivity of CD4^+^ T cells by reducing the activation of protein kinase C-α via CD44. These results suggest that CD44–HA interactions are important for HIV infection and that addition of exogenous HA may interfere with this interaction. Furthermore, this inhibition of infectivity by exogenous HA may be mediated by the inhibition of the protein kinase C-α pathway and is dependent on the thickness of HA at the cell surface. It would be interesting to see if these results can be replicated *in vivo*, and whether the reduction of infection is dependent on the average molecular weight of exogenous HA and/or HA at the cell surface.

The expression of gamma interferon-inducible protein 10 (IP-10) has been shown to be elevated in chronic HCV patients and is a predictor of treatment outcome (40). In a study aimed at elucidating what role TLRs play in the production of IP-10 in cells infected by and actively replicating HCV, CD44–HA signaling was shown to be involved in IP-10 production (41). In this study, CD44 expression was found to be increased in cells harboring viral replicons. Blocking viral replication led to a reduction in CD44 expression. Furthermore, HA stimulation of cells with actively replicating HCV virus increased IP-10 production; contrarily, knockdown of CD44, TLR2, or MyD88 greatly reduced IP-10 production. These results suggest that in infected cells, HCV induces IP-10 production via CD44–TLR2–MyD88 interactions. This study also links HCV replication to increased expression of CD44. An interesting follow-up study may elucidate whether HCV replicon mediated up-regulation of CD44–HA signaling and increased production of IP-10 leads to leukocyte recruitment and the establishment of chronic inflammation in the liver, leading to HCV-associated fibrosis, cirrhosis, and hepatocellular carcinoma.

These studies further illustrate the complex roles that CD44–HA signaling plays in human infections by pathogens. In the case of HIV infection, it appears that exogenous and endogenous HA protect host cells from infection in a CD44-dependent manner. The opposite is seen in the case of HCV infections; overexpression of CD44 by HCV replicating cells may contribute to overproduction of IP-10, exacerbation of liver damage, and to a poor treatment outcome in chronic HCV patients.

### Interstitial lung disease

CD44–HA signaling has been implicated in the progression of ILD, which includes idiopathic pulmonary fibrosis (IPF) and systemic sclerosis associated ILD. CD44–HA signaling was shown to play a role in the development of progressive lung fibrosis by activation of myofibroblasts with an acquired invasive phenotype (42). In this study, HAS2-overexpressing transgenic mice showed increased deposition of HA and accumulation and up-regulation of CD44 mRNA and protein in lung myofibroblasts after bleomycin-induced lung injury, resulting in increased lung fibrosis and fatality. However, HAS2 knockout mice did not have increased HA deposits, up-regulation of CD44 or myofibroblast accumulation, and did not show signs of pulmonary fibrosis. Interestingly, the study also found that fibroblasts isolated from HAS2-overexpressing transgenic mice showed increased invasive capacity in Matrigel ™when compared to controls and HAS2-null mice. This increase coincided with increased matrix metalloproteinase (MMP) levels and decreased tissue inhibitors of metalloproteinases levels in HAS2-overexpressing transgenic mice and also in the fibroblasts from IPF patients. The results from this study suggests that HAS2 overexpression, post-lung injury, leads to dysregulated CD44–HA signaling, resulting in a signaling environment conducive to fibroblast activation and invasion leading to the development of IPF. It would be important to determine if this HA-mediated signaling environment is dependent on the molecular weight of HA. For example, addition of exogenous HA of specific mass to isolated IPF fibroblasts may further elucidate the nature of CD44–HA signaling in progression of lung fibrosis. Another important study would be to evaluate the respective roles of CD44 and receptor for hyaluronic acid-mediated motility (RHAMM), which are expressed on fibroblasts (43), in the development of IPF.

A recent study evaluated the relationship and function of CD44v6, TGF-β1, and hepatocyte growth factor (HGF) in ILD (44). The study found that lung fibroblasts from bleomycin-treated mice and from ILD patients had elevated levels of CD44v6 and c-Met, both of which were expressed upon TGF-β1 induction. CD44v6 was shown to increase collagen-1, and therefore, enhance lung fibrosis. TGF-β1 induction and lung fibrosis were shown to be abolished by HGF. One caveat of this study is that HGF levels are increased in fibroblasts from ILD patients, which is contradictory to the finding that HGF negatively regulates TGF-β1 induction, and therefore inhibits lung fibrosis. However, HGF concentrations in ILD fibroblasts were much lower than the concentrations used in the study.

In summary, these studies reveal a causative role of CD44 and its isoform CD44v6 in ILD. Furthermore, HA is important in the progression of fibroblast invasion and lung fibrosis. Interestingly, a recent study found that disruption of the HA matrix or inhibition of HA synthesis actually increased deposition of fibronectin and collagen-1 by myofibroblasts in which TGF-β1 was induced, and HAS2 expression was increased in these myofibroblasts (45). These results together suggest that disruption of endogenous HA or tissue matrix homeostasis may cause dysregulated deposition of fibronectin and up-regulation of HA by fibroblasts that have constitutively active TGF-β1 signaling. Up-regulation of HAS2 expression in these fibroblasts leads to aberrant CD44–HA signaling, which may lead to activation of more fibroblasts with invasive phenotypes.

### Wound healing

Hyaluronic acid concentrations in tissues undergoing wound repair are elevated for several weeks after injury (46). The primary source of this HA is keratinocytes, but other tissue specific cells can produce the glycosaminoglycan as well. One of the functions of HA in wound repair is to provide a scaffold for cell migration toward injury sites such as hepatic stellate cells migrating toward a liver epithelial injury utilizing CD44v6 (47).

A recent study in a rat bladder regeneration model found that HA deposition in a regenerating bladder after partial cystectomy was required for proper wound healing (48). CD44 expression also increased as the wound healed, mimicking the deposition pattern of HA. The function of the HA–CD44 interaction could simply be to provide a scaffold for tissue repair, but other research shows that LMM and HMM HA found in sites of injury may have direct effects on regeneration. In a mouse acute myocardial infarction model, it was found that IL-6 production in the heart induced HA synthesis, and that HA was required for the differentiation of fibroblasts into myofibroblasts (49). HA binding to CD44 also leads to the production of MCP-1 and CCL5, which recruit neutrophils to the heart and establish a cardio-protective environment. It has previously been shown that CD44 and ERK1/2 complexes exist in fibroblasts and are required for scratch repair (43).

It is not just the presence of HA and CD44 that shape the wound healing process, but also the size and longevity of the HA fragments. HMM HA (>10^6^ Da) is typically present in prenatal wound healing, resulting in healing with minimal inflammation and scar formation (50–53). HA oligosaccharides (six to eight oligomers) are typically present in adult wound healing, and results in inflammation and scar formation (54–57). If HA oligomers are not removed from tissue during wound repair then tissue destruction occurs from unending fibrosis and inflammation (21). The negative impact of prolonged accumulation of LMM HA on wound repair is primarily caused by its ability to bind to and activate TLR4. In a mouse sterile injury model, it was found that MD-2, a TLR4 accessory protein that increases TLR4’s ability to bind lipopolysaccharide, and CD44 worked together to bind LMM HA on monocytes (21). This prolonged TLR4 signaling-induced production of TGF-β2 and MMP-13, resulting in tissue damage much the same as if lipopolysaccharide was present in the wound. This finding expands upon earlier research showing that CD44 can associate with MyD88 signaling complexes and TLRs in lipid rafts (58).

A recent study using normal human dermal fibroblasts also shows that LMM HA (4.3 kDa) induces the production of IL-6, IL-8, CXCL1, CXCL2, CXCL6, and CCL8 whereas, HMM HA (1.1 × 10^6^ Da) can inhibit the production of IL-6 and IL-8, indicating potential TLR and MyD88 involvement (59). Closer examination of the effect of specific sizes of HA has revealed that six oligomer fragments of HA are capable of promoting wound closure, accumulation of M2 macrophages, and production of TGF-β1 without inducing myofibroblast differentiation in a scratch wound repair model (60). Conversely, HA fragments of 40 kDa were found to actively inhibit wound closure and cause the accumulation and differentiation of myofibroblasts.

In conclusion, HA binding to CD44 is required for proper wound healing, but the size and longevity of the HA has a large effect on the outcome. Future research on the size and location of exogenous HA that can be used to aid in wound healing will be of great medical benefit. Surgical scarring could be a thing of the past if small oligomers of HA could be introduced into a wound after incision, but the fragments would need to have a short biological half-life in order to prevent fibrosis and inflammation from occurring.

## CD44–HA Signaling and Cancer

CD44 signaling has been shown to be important in cancer metastasis and tumor growth, but can be broken down into two primary areas. HA-independent signaling relies on interactions between CD44’s intracellular domain and cytoskeletal proteins or membrane associated kinases. HA-dependent signaling relies on two CD44 molecules being cross-linked, allowing other CD44-associated signaling proteins to interact with each other. Further, CD44 is used as a marker for cancer stem cell (CSC) detection in a variety of cancers without much research into the function of CD44 in stem cells beyond adhesion.

### HA-independent signaling

CD44 signaling that is HA independent is largely reliant on CD44’s position in lipid rafts. Association between CD44 and other signaling proteins are often regulated through CD44’s interaction with ezrin–radixin–moesin (ERM) protein family members that initiate association with cytoskeletal elements.

A recent study examines the role of CD44 in Wnt pathway signaling using *Xenopus* embryos (61). In this study, CD44 was found to be a positive regulator of Wnt pathway activation by associating with LRP6. In this model, a comparison between various CD44 splice variants showed that all isoforms of CD44 are capable of association with LRP6, but some isoforms such as CD44v6 may be associated with higher levels of Wnt activity. The Schmitt study also shows that while the extracellular domain of CD44 interacts with LRP6, the intracellular domain interacts with and coordinates a protein complex with the rest of the Wnt pathway downstream targets through cytoskeletal arrangement. HA-independence was verified in this study by utilizing hyaluronidase treatment and an anti-CD44 antibody that blocks the HA binding domain. This recent finding is significant because CD44 variant isoform expression is correlated with tumor progression in colorectal cancer and high Wnt activity, which is the signaling pathway known to control colorectal cancer progression (62–64).

A similar signaling pathway is found in hepatoma, colon adenocarcinoma, and cervical carcinoma cells, which results in the ability to scatter and spread (65). The Orian-Rousseau study illustrates the fact that CD44v6 is required for c-Met activation upon HGF stimulation. The extracellular domain of CD44v6 is required for autophosphorylation of c-Met, but the intracellular domain is required for further downstream signaling. The interactions between CD44’s intracellular domain and ERM protein family members facilitate the association of GRB2 and SOS. In this way, CD44v6 serves as a linkage between the cell surface c-Met protein and the MAPK/ERK pathway members that are associated with the cytoskeleton. Recently, it was discovered that CD44v6 is also required for internalization of activated c-Met so that c-Met can be recycled or degraded (66). Hasenauer et al. showed that the Met receptor was linked to the actin cytoskeleton via CD44 and Ezrin, which allowed for endocytosis of activated Met in order to sustain regulated cell migration and branching. ERM protein family interaction with CD44 and the cytoskeleton can be disrupted by decreasing the amount of available phosphatidylinositol 4,5-bisphosphate (67). This finding can be exploited depending on the context through activation or inhibition of phospholipase C proteins, which decrease the amount of phosphatidylinositol 4,5-bisphosphate available upon activation.

Another recent novel finding is the association of CD44 with PP2A, a phosphatase known to dephosphorylate Raf, MEK, and Akt (68). In this study, EL4 T cell lymphoma cells were found to show decreased ERK phosphorylation and increased apoptosis upon ligation of CD44 with an anti-CD44 antibody that also blocks the HA binding domain. The CD44 ligation induced the binding of PP2A to the intracellular domain of CD44 as well as PP2A’s activation. The activated PP2A then dephosphorylates ERK1/2 without causing its degradation, resulting in activation of the mitochondrial death pathway. It is not known if the interaction of PP2A and CD44 requires the ERM protein family or cytoskeleton.

In summary, CD44 standard and variant isoforms are closely associated with many signaling pathway proteins that drive tumor development and progression in many types of cancer. These associations exist and activate signaling cascades without direct binding of CD44 to its ligand, highlighting the importance of CD44 as a co-receptor and emphasizing its importance and potential in targeted cancer therapy. The findings discussed above may not tell the complete story of HA-independent CD44 signaling. Experiments often rely on antibodies against the HA binding domain of CD44 and hyaluronidase treatment to study HA-independent CD44 signaling, but it is not possible to completely eliminate endogenous HA. Hyaluronidase treatment alone may simply cleave HA within the extracellular matrix and induce oligomer binding to CD44. Blocking antibodies compete with endogenous HA for receptor binding, and may not completely prevent HA binding; these antibodies may also cross-link CD44 on the cell surface in a similar manner to HA binding, resulting in a phenomenon that could be observed with exogenous HA. Truncated CD44 expression or use of CD44 knockout mice are two potential ways to verify HA-independence, but this proves a complicated feat to perform in cell lines that already express high levels of CD44 and human samples.

### HA-dependent signaling

**Figure 1:**
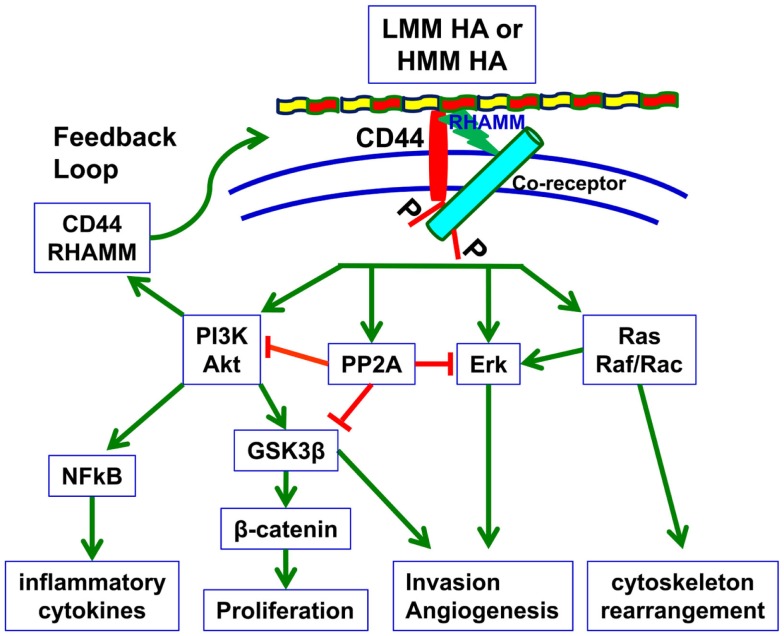
**HA-dependent CD44 signaling**. HA binding to cell surface CD44 and RHAMM triggers a variety of signaling events, including complex formation between CD44 and co-receptors such as c-Met, EGFR, and TGF-β receptors, and activation of downstream effectors such as Akt, PI3K, PP2A, ERK1/2, and Ras/Raf/Rac. These signaling events culminate in the expression of a variety of inflammatory cytokines and activation of a feedback loop continuing cell surface expression of CD44/RHAMM. By inducing these signaling events and downstream effectors, HA–CD44 signaling drives proliferation, invasion, cytoskeletal rearrangement, and angiogenesis, which lead not only to normal cell functions such as fibroblast migration, wound healing, and immune cell function but also to tumor growth and progression (70).

CD44 does not exist without ligand binding capabilities, and the interaction between CD44 and its major ligand HA induces signal transduction as well (Figure 1). HMM HA (>950 kDa) has been shown to promote proliferation of decidual cells during the early stages of pregnancy by activating the PI3K/Akt and ERK1/2 signaling pathways (69). Several recent studies have also focused on the function of CD44 isoforms in various types of cancer upon the addition of HA.

A recent study has shown that when fibroblasts are allowed to adhere onto a surface coated in HA; CD44, CD36, PP2A, and CDK9 are upregulated (71). PP2A expression increases in these fibroblasts, mimicking what was seen in T cell lymphoma in the Rajasagi study. The Yang study shows that natural conditions resulting from HA polysaccharides binding and cross-linking CD44 on fibroblast surfaces increase the expression of PP2A. The Rajasagi paper demonstrates PP2A association with CD44 through therapeutic intervention with anti-CD44 antibodies and no exogenous HA. One of the more thoroughly investigated aspects of HA-dependent CD44 signaling is in its role in facilitating epidermal growth factor receptor signaling.

The above two studies illustrate the fact that the signaling pathways seen in HA-dependent CD44 signaling are almost identical to those seen in HA-independent signaling. CD44 isoforms are associated with downstream pathway molecules such as PI3-kinase, SRC, SOS, and GRB2 through the ERM family (65, 72–74). This association allows for CD44 in lipid rafts to essentially bring all of the necessary signaling components of multiple pathways with them. When HA binding to CD44 occurs it may bring CD44 in close proximity to signaling receptors such as ErbB2, EGFR, or TGF-β type 1 receptors, allowing for direct association and interaction between receptors and their signaling complexes in a tightly localized lipid raft (75–78).

Recent work has highlighted novel areas of CD44–HA interaction. A study in MDA-MB-468 breast cancer cells has shown that CD44 cross-linking and signaling upon HA binding activates c-Jun nuclear translocation (79). Activated c-Jun induces the transcription of microRNA-21, which increases the amount of Bcl-2 and upregulates inhibitors of the apoptosis family of proteins. The HA-dependent CD44 signaling also induces chemoresistance. This finding can help transition CD44 research from the more classical viewpoint that it serves as a marker without much functional role into a biomarker that has a known and exploitable function.

CD44 does not bind to HA alone. RHAMM also binds to HA in a complex with CD44 in order to facilitate signal pathway involvement. In prostate cancer cells, HA and HA fragments were shown to induce growth factor receptor signaling, including the PI3K/Akt pathway (80, 81). However, knockdown of both RHAMM and CD44 with siRNAs was required to achieve complete inhibition of HA-mediated signaling events. RHAMM, CD44, and ERK1/2 are in a signaling complex and can be co-immunoprecipitated from MDAMB231 and Ras-MCF10A breast cancer cells; this complex promotes sustained high basal motility (82). The Hamilton study also used anti-RHAMM antibodies to block MEK activation, illustrating the important of RHAMM as a co-receptor with CD44. RHAMM has also been shown to be required for CD44–HA signaling in head and neck cancer and cementifying fibroma (77, 83).

## CD44 as a Cancer Stem Cell Marker

CD44 has lately emerged in CSC or cancer initiating cell studies as a biomarker. A large number of CSC studies use CD44 standard or variant isoform expression as a typical marker (84–87). Current research is focusing on the role of CD44 signaling during epithelial–mesenchymal transition (EMT).

A recent study using mouse pancreatic tumor cells shows that CD44 is expressed on a specific population of pancreatic cancer cells (88). CD44 expression drives up-regulation of Snail1, resulting in increased MT1-MMP, which facilitates invasion. MT1-MMP is also known to directly associate with CD44 at the cell surface, where CD44 aids in the recycling of MT1-MMP so that cells can continue to degrade the extracellular matrix (89, 90).

An in-depth look at current literature for CD44 and EMT reveals that most studies use CD44 as a marker instead of a potential mechanism. A recent study shows that microRNA-106b is upregulated only in CD44^+^ gastric cancer cells undergoing EMT and that TGF-β signaling was also elevated (91). Coupled with the established link between CD44 expression and other microRNAs, as well as the relationship between CD44 and TGF-β, one could make the case for further study into the potential for CD44 expression to be the cause of this phenomenon rather than a simple marker for EMT. A recent study of a mouse mammary epithelial cell line (NMuMG) and a triple negative human breast cancer cell line MDAMB231 shows that CD44 couples with VCAM-1 in order to initiate EMT as well as chemoresistance (74). Physical association between VCAM-1 and CD44 resulted in increased expression of ABCG2, a drug efflux pump responsible for inducing resistance to chemotherapy (92).

A recent study of human primary prostate cancer tissues and lymph node metastases revealed that CD44v6 was highly upregulated in tumors and CSCs (93). Knockdown of CD44v6 with siRNA in DU145, PC3M, and LNCaP cells increased sensitivity to doxorubicin, methotrexate, docetaxel, and paclitaxel. CD44v6 knockdown also reduced tumor sphere formation and proliferation. The Ni study also showed that CD44v6 was responsible for activation of PI3K, Akt, mTOR, and Wnt signaling pathways, which were driving EMT in these cell lines.

A study by Kinugasa et al. also showed that CD44 was important for maintaining the stemness of cancer cells, but in an indirect manner (94). Cancer-associated fibroblasts from B16 melanoma tumors were found to express high levels of CD44, and co-culture of human colorectal cancer HT29 cells and human lung carcinoma LLC1 cells with these fibroblasts resulted in drug resistance, tumor sphere formation, and tumor growth. Cancer-associated fibroblasts generated from CD44 knockout mice did not result in a sustained CSC state. The pathways regulated by CD44 in these fibroblasts have yet to be determined, but the studies from Ni and Kinugasa illustrate the complex nature of CD44 signaling in cancer.

Several studies do not directly examine the role of CD44 in CSCs, but focus on its absence or presence in response to gene silencing and other treatments. When STAT3 is knocked down in MCF7–HER2 breast cancer cells, the expression of CSC markers CD44, Oct-4, and Sox-2 was downregulated, resulting in decreased tumor sphere formation (95). Potential CD44–PI3K and CD44–ERK signaling would be eliminated in the case of CD44 downregulation, which could explain the absence of tumor sphere formation in these cells. Treatment of human T24-L bladder cancer cells with Silbilin results in a decrease in CD44 expression, spheroid colony formation, side population presence, and a reversal of EMT (96). Silbilin also inhibited β-catenin and ZEB1 signaling, possibly through inhibition of GSK3β phosphorylation. As discussed above, CD44 is involved in the Wnt pathway and therefore β-catenin, but CD44 can also positively and negatively regulate GSK3β (83, 97). Therefore, CD44 may be a central molecule in several signaling pathways, but many studies still examine it only as a marker of stemness.

In conclusion, changing the perspective of CD44 expression from that of a simple marker to a protein, which causes cancer growth and progression, will pave the way for future therapeutic intervention. The studies highlighted above that focused on the role of CD44 as a signaling molecule rather than a marker without a function, are a fragment of the potential research yet to be unlocked. Continued discovery of novel roles for CD44 in cancer development will help expand the mounting potential of CD44 as a prime therapeutic target.

## Targeting HA Receptors

Since the discovery that HA receptor, CD44 is a stem cell marker, targeting of CD44 for anti-cancer therapy has been attempted using DNA vaccines, anti-CD44 monoclonal antibodies, and nanoparticle-mediated delivery of CD44siRNAs. In addition, HA-coated nanoparticles or anti-CD44 conjugates have been used to target CD44^+^ cells for therapy.

### CD44 vaccines

CD44 cDNA or targeting of CD44-expressing cells has been used to generate tumor immunity in experimental models. A recent study has examined the concept of “foreignizing” tumor cells by specifically delivering foreign antigens to target CD44^hi^ tumor cells using a polymeric ovalbumin (foreign antigen) and HA delivery system (98). In this study, the polymeric conjugate was accumulated on CD44^hi^ tumor cells. Furthermore, the surface class I MHC antigens on these tumor cells displayed an OVA_257–264_ peptide. When these tumor cells were injected in mice, which were immunized with a vaccinia virus expressing ovalbumin, tumor growth was reduced due to OVA_257–264_ peptide specific cytotoxic T-lymphocytes. CD44 cDNA vaccination also has been delivered by implanting virtual lymph nodes in immunocompetent animals to generate anti-CD44 antibodies. These virtual lymph nodes are generated by subcutaneous injection of a silicon tube filled with a segment of hydroxylated-polyvinyl acetate wound dressing sponge in which CD44-standard or CD44-variant cDNA is inserted. Using this model, CD44-standard form vaccination was shown to reduce autoimmune encephalomyelitis (99).

In a similar approach of using virtual lymph nodes, the effects of CD44 vaccination on tumor growth and lung metastasis were evaluated in a mouse mammary adenocarcinoma model (DA3 cells). Vaccination was achieved by injection of virtual lymph nodes loaded with human CD44 variant (v3–10) or CD44-standard cDNAs. Immunized animals expressed antibodies against human CD44 variant and CD44-standard forms. The vaccination against CD44 variant (v3–10) was more effective than vaccination with the CD44-standard isoform in eliminating tumor growth in 75% of the vaccinated mice and slowed tumor growth in the remaining animals. Furthermore, metastasis was eliminated in all animals. Since CD44-standard form did not generate the same immunological response against tumors, it suggested that CD44 variant (v3–10) and not CD44s was functional in promoting tumor growth and metastasis in DA3 cells. It is noteworthy that in this study human CD44 (hCD44) was injected in order to break the tolerance to mouse CD44 (mCD44). Therefore, mouse CD44 (mCD44) should be injected to human patients to break CD44 tolerance, but this is not practical in clinical setting (100). The virtual lymph node and CD44 cDNA approach has also been used to induce resistance to insulin-dependent diabetes. In this study, both CD44 standard and CD44v3–v10 cDNAs induced resistance to diabetes to the same extent, and the resistance was antibody mediated (101). As in the study by Wallach-Dayan et al., this study also used human CD44 cDNA to break the self-tolerance to mouse CD44, and the use of mouse CD44 cDNA did not generate a humoral response. This may be a likely reason as to why the virtual lymph node approach, involving CD44 cDNA vaccination has not been translated into clinical trials. Another approach for CD44 vaccination involves dendritic cell vaccination. In the B16 melanoma lung metastasis model, dendritic cells were pulsed with anti-CD44 coated apoptotic B16 melanoma cells. Such opsonized B16 melanoma cells were readily endocytosed by dendritic cells. Following vaccination of mice with dendritic cells, animals were challenged with subcutaneous injection of B16 cells. In vaccinated animals, both lung metastasis (50% reduction) and tumor growth were inhibited. Moreover, 60% of the animals remained tumor-free for 8 months. In this model, vaccination-induced B16 cell-specific CD8 T cells (102).

In summary, although the high expression of CD44 in CSCs, and other cell types render CD44 as an attractive molecule for a targeted vaccine therapy, the use of CD44 vaccination has been confined to pre-clinical studies in very limited number of cancer and non-cancer models.

### CD44 siRNA delivery

In a few studies, CD44 has been targeted for therapy using specific siRNAs. A challenge with this approach is the alternatively spliced isoforms of CD44. Based on the sequence, the siRNAs may downregulate only certain CD44 variants. The most common isoforms targeted by siRNAs are CD44-standard and CD44v6. Most commonly these CD44 siRNAs have been delivered to tumor cells using nanoparticles. For example, biodegradable poly d,l-lactide-co-glycolide acid nanoparticles have been used to simultaneously deliver CD44 and FAK siRNAs to ovarian cancer xenografts. Knockdown of both genes reduced tumor growth by inhibiting angiogenesis and proliferation index in tumors (103). More recently, a nanoscale-based drug delivery system was tested to inhibit the growth of an ovarian cancer xenograft model. The nanoscale delivery system contained a modified polypropyleniminedendrimer as a carrier, paclitaxel, a synthetic analog of luteinizing hormone-releasing hormone peptide for targeting tumor cells, and siRNA targeted to CD44 mRNA. This dendrimer was able to downregulate CD44 mRNA and protein expression and inhibit tumor growth without toxicity, suggesting that the targeted delivery of CD44 siRNA along with chemotherapeutic agents may be explored for cancer therapy (104). However, this study did not specify which CD44 isoforms were targeted by the siRNAs. Delivery of CD44 siRNAs by coating them on microneedles for self-delivery was shown to reduce the expression of CD44 in human skin xenografts in immunocompromised mice (105). Similarly, a CD44v6 targeting siRNA encapsulated into polyethylene glycol-poly-l-lysine micelles was shown to accumulate into tumor tissues and reduces tumor growth in a pancreatic xenograft model (106).

### Targeting CD44 for delivering antitumor therapies

Since CD44 is overexpressed in a variety of tumor cells, HA-coated self-assembling nanoparticles or liposomes have been tested for the delivery of siRNAs and/or chemotherapy drugs in pre-clinical xenograft models. The siRNAs reported so far include those specific for the multidrug resistance (MDR) protein or proteins in the apoptosis pathway (e.g., bcl-2, survivin). The advantage of CD44-targeting HA-coated nanoparticles for siRNA delivery is that these nanoparticles are biodegradable and reasonably specific to tumors. For the proper function, encapsulation and stabilization of HA-coated nanoparticles, HA has been combined with various materials including, poly-l-lysine-graft-imidazole-based polyplexes (107), lipids of varying carbon chain lengths/nitrogen content and polyamines (108), protamine sulfate interpolyelectrolyte complexes (109), near infrared dyes for imaging (110), chitosan(CS)-triphosphate (111), poly(dimethylaminoethyl methacrylate) for cross-linking of siRNAs (112) and polypropylenimine dendrimer (104). Some studies have confirmed that the uptake of the HA-coated nanoparticles or liposomes by tumor cells is mediated by HA-receptor mediated internalization. In these studies, incubation of tumor cells with soluble HA inhibited uptake of these materials and non-CD44-expressing cells did not show preference in up-taking the HA-coated liposomes when compared to the control liposomes (110, 113).

The efficacy of different HA-coated nanoparticles to deliver siRNAs has been studied in xenograft models, where the preferred model is a subcutaneous implantation of tumor cells and intravenous delivery of the nanoparticles. For example, HA-poly(ethyleneimine)/HA-poly(ethylene glycol) has been used to deliver MDR1 siRNA to OVCAR8TR (established paclitaxel resistant) tumors. Following the downregulation of P-glycoprotein, these xenografts become sensitive to paclitaxel treatment (114). HA nanoparticles have been loaded with a near infrared dye (amphiphilic carbocyanine dye that strongly absorbs and fluoresces in the near infrared region) to visualize the distribution of HA-coated nanoparticles in various tissues, by live animal imaging. In this study, intravenous delivery of the nanoparticles and imaging showed that HA-cisplatin nanoparticles had favorable safety profile for targeted delivery of cisplatin to tumors, while the HA-poly(ethyleneimine)/HA-poly(ethylene glycol) nanoparticles were efficient in delivering the siRNAs (e.g., survivin) to cisplatin resistant but CD44-overexpressing tumors (110).

In the nanoparticles that deliver the siRNAs, polymeric compounds (e.g., polyethyleneimine or polyethyleneglycol) form ionic complexes with the siRNAs; however, these nanoparticles are not stable. To improve stability while retaining biodegradability, a HA-graft-poly(dimethylaminoethyl methacrylate) nanoparticle has been designed. In this nanoparticle, siRNAs are cross-linked via a disulfide linkage (112). These nanoparticles were shown to efficiently accumulate in the CD44-overexpressing murine melanoma tumor tissues and the cross-linked siRNAs had 50% more stability than the uncross-linked siRNAs. One study has designed nanoparticles involving HA and protamine sulfate interpolyelectrolyte complexes for delivering miR-34a to breast cancer cells *in vitro* and in the MDAMB231 subcutaneous xenografts. The nanoparticle delivery of miR-34a reduced tumor growth by 70% and showed TUNEL positive cells in tumor tissues (109).

For the delivery of anti-cancer drugs such as doxorubicin, a photochemically triggered cytosolic drug delivery system based on combining pH-responsive HA nanoparticles containing doxorubicin has been developed (98). The pH responsiveness of these nanoparticles leads to doxorubicin release, and results in significant antitumor efficacy both *in vitro* and *in vivo*. Similarly, paclitaxel loaded hyaluronate-cholanic acid nanoparticles with FlammaTM-774 fluorescent dye imaging have been used to track the targeting of these nanoparticles to tumors upon intravenous injection (115). The nanoparticles showed accumulation and retention up to day 6 in tumors (SCC7 squamous cell carcinoma model) upon intravenous injection, with little accumulation in other organs. Furthermore, the Paclitaxel loaded nanoparticles decreased tumor growth by over 60%, while free Paclitaxel at the same concentration (5 mg/kg) had little effect on tumor growth. Amphiphilic cholesteryl-succinoyl hyaluronan (Chol-Suc-HA) conjugates self-assembled into docetaxel-loaded nanoparticles in the aqueous environment have been evaluated both *in vitro* and *in vivo*. While all docetaxel-loaded Chol-Suc-HA nanoparticles showed high drug loading, uniform particle size distribution and stability *in vitro*, nanoparticles with higher degree of substitution of the hydrophobic moiety had significantly more stability in plasma and antitumor efficacy in breast cancer xenografts (116). Similarly, mTOR inhibitor rapamycin chemically conjugated to HA nanoparticles via a novel sustained-release linker, 3-amino-4-methoxy-benzoic acid was found to slow down the clearance of rapamycin by 8.8-fold in immunocompetent mice bearing CD44-positive 4T1.2neu breast cancer cells. In this pre-clinical model, rapamycin conjugated HA nanoparticle inhibited tumor growth and increased survival (117).

Hyaluronic acid-coated magnetic nanoparticles have been tried for the delivery of chemotherapeutic drugs to tumors. An advantage of these nanoparticles is their tracking by magnetic resonance imaging. In an ovarian cancer model, the HA-coated superparamagnetic iron oxide nanoparticles laded with doxorubicin delayed, as well as, reduced tumor growth and increased survival (118).

In summary, HA-coated nanoparticles can be conjugated to a variety of materials for the delivery of siRNAs or chemotherapy drugs. Although it is clear that the nanoparticles are taken up by tumor cells or tissues via receptor-medicated endocytosis, it is less clear whether the nanoparticles are taken up only through CD44-medidated endocytosis. This is because a variety of tumors also overexpress RHAMM. Since in most studies, the efficacy of these nanoparticles has been evaluated in immunocompromised mice, it is unclear what the distribution will be of these nanoparticles in an immunocompetent host. The latter is of importance since CD44 was originally known as a “lymphocyte homing receptor” and is highly expressed in both B- and T-lymphocytes. However, since the major (and probably the only) CD44 isoform expressed in lymphocytes is the standard form, siRNAs specifically directed to the variant isoforms should have the specificity for targeting tumors. Nevertheless, the long-term toxicity, stability, and tissue distribution of the various HA-coated nanoparticles will need to be evaluated in relevant hosts before these nanoparticles can be tested in clinical trials.

### Targeting of CD44 protein

The efficacy of anti-CD44 antibodies has been evaluated in murine models of autoimmune and inflammatory diseases including thrombocytopenia and arthritis. Immune thrombocytopenia is an autoimmune bleeding disorder characterized by a low platelet count and the production of anti-platelet antibodies (119, 120). While the standard treatment for immune thrombocytopenia is passive infusion of immunoglobulins, several anti-CD44 antibodies have been shown to ameliorate immune thrombocytopenia. However, some anti-CD44 antibodies (i.e., IM7, IRAWB14.4, 5035-41.1D, KM201, KM114, and KM81), which reduce serum-induced arthritis can themselves induce thrombocytopenia in murine models (120). It has been suggested that since CD44 is not expressed in human platelets, anti-CD44 antibodies should not induce thrombocytopenia in patients. A fully humanized rat anti-CD44 monoclonal antibody, PF-03475952 was found to cause a dose-dependent decrease in symptoms in a mouse model of collagen-induced arthritis. This monoclonal antibody was found to be safe in pharmacological assays. The antibody was suggested as a treatment for inflammatory diseases such as rheumatoid arthritis (121); however, no further studies were conducted on this antibody and the antibody does not appear to have entered in clinical trials. In a genome-wide association study, CD44 was found to be a functionally associated gene in type 2 diabetes patients (122). The same study found that intraperitoneal administration of an anti-CD44 antibody in a murine model of high fat diet induced type 2 diabetes, decreased blood glucose levels, and macrophage infiltration in adipose tissues (123). Furthermore, daily injection of the anti-CD44 antibody decreased blood glucose levels, weight gain, liver steatosis, and insulin resistance to the levels lower than anti-diabetes drugs metformin and pioglitazone (122).

Anti-CD44 antibodies have also been evaluated as an anti-cancer therapeutic. In chronic lymphocytic leukemia cells, which express high levels of CD44, a humanized monoclonal antibody specific for CD44 (RG7356) was found to be cytotoxic to leukemia B cells without affecting the viability of normal B cells. Systemic administration of this antibody caused complete clearance of leukemia xenografts. Interestingly, the effects of the antibody were not neutralized in the presence of HA, suggesting that CD44 may have functions other than binding to HA, which play a role in leukemia (124). The bio-distribution of the same antibody has been evaluated in the CD44(+) and CD44(−) xenograft bearing mice and normal cynomolgus monkeys, following radiolabeling with (89)Zr (125). The study found that while the uptake of the (89)Zr-RG7356 antibody in CD44^+^ xenografts was ~9-fold higher than in CD44(−) xenograft, the uptake in CD44(+) xenograft was similar, regardless of whether the xenograft was responsive or non-responsive to the anti-CD44 antibody. Similarly, the antibody was detected in the spleen, salivary glands, and bone marrow, most likely because these organs express high levels of CD44 (125). Therefore, for targeting tumors a large dose of an anti-CD44 antibody might be needed and furthermore, simply the presence of the antibody in tumors may not be indicative of its efficacy. In pancreatic cancer, where CD44 expression correlates with poor prognosis, intravenous delivery of an anti-CD44 monoclonal antibody H4C4 has been shown to completely inhibit tumor growth and metastasis in two different pancreatic xenograft models. In the same study, H4C4 was also able to eliminate tumor initiating cells, as well as, tumor recurrence following radiation treatment. This suggests that targeting of CD44-overexpressing pancreatic CSCs may improve outcome in pancreatic cancer patients who undergo radiation therapy (126).

In addition to the therapeutic uses of various anti-CD44 antibodies, a study has used an anti-CD44 antibody for tumor imaging. For example, a chimeric monoclonal antibody U36 and its F(ab′)2 and Fab′ fragments that recognize the CD44v6 isoform have shown potential for radioimmuno-therapy and radioimmuno-targeting of experimental tumors (127, 128). A ^99m^Tc-labeled U36, when used in conjugation with a single-photon emission computed tomography has been shown to detect all primary tumors of head and neck squamous cell carcinoma. In clinical trials, this antibody labeled with ^186^Re-labeled could detect up to 66% of breast cancer lesions (129). However, these techniques do not visualize micro-tumors, tumor nodes with necrosis, or tumors containing keratin or fibrin. Another humanized anti-CD44v6 antibody VFF18 labeled with a near infrared dye IRDye800Cw has shown promise in tracking ductal carcinoma *in situ* in mouse xenografts (130).

As in the case of HA-coated nanoparticles or liposomes, anti-CD44 antibody–drug conjugates have also been used either for imaging of tumors or for delivering chemotherapeutic agents to experimental tumor models. For example, anti-CD44 antibody conjugates have been used to deliver radioisotopes or mertansine for the treatment of CD44-expressing tumors. In these studies, disease stabilization was observed in breast or head and neck tumor patients; however, dose-limiting toxicity was observed along with the distribution of the antibody in the skin, where high levels of CD44 are expressed (131). In phase I studies, maximum tolerated dose, safety, and efficacy of an immuno-conjugate BIWI 1 (bivatuzumabmertansine), consisting of a highly potent anti-microtubule agent coupled to an anti-CD44v6 monoclonal antibody, was evaluated in head and neck cancer patients. In this study, while three patients showed a partial response, the binding of BIWI to CD44v6 on skin keratinocytes caused serious skin toxicity with a fatal outcome, leading to early termination of this trial (132). More recently, nanoparticles and liposomes containing an anti-CD44 antibody, as well as, imaging reagents (e.g., cDNAs for monomeric red fluorescence protein or luciferase) have been used to target tumor detection and imaging (86). Anti-CD44v6 single chain variable fragment [scFv(CD44v6)] screened out from the human phage-display library has been used for the targeting of arsenite ion (As) encapsulated nanoparticles to CD44-positive cells. Upon intravenous delivery, these nanoparticles specifically accumulated in the PANC-1 tumor xenografts for up to 2 days and completely inhibited tumor growth (133). However, none of these reagents have been evaluated in clinical trials.

The HA binding domain is highly conserved in all CD44 isoforms and, therefore, attempts have been made to target this domain using specific mono-thiophosphate-modified aptamers. These aptamers bind specifically to CD44-expressing tumor cells with high efficacy. However, the *in vivo* bioavailability of these aptamers has not been examined in detail (134).

In summary, targeting of CD44 by siRNAs or antibodies for therapy and the use of HA-coated nanoparticles or of anti-CD44 antibody conjugates for the delivery of therapeutic agents have been examined as attractive strategies in the treatment of cancer and chronic diseases. In addition, anti-CD44 antibodies have also been used for imaging purposes. However, the majority of the studies are limited to pre-clinical models. In limited clinical studies, the use of anti-CD44 antibodies has resulted in significant toxicity. In addition, the HA-coated nanoparticles may also deliver the cargo to tissues, which express other HA receptors. Therefore, treatment and imaging strategies that target CD44 will have to be carefully evaluated for their effects on normal cells, and the immune system. Furthermore, the risk versus benefit must be carefully evaluated before CD44-targeting strategies are translated to the clinic.

## Conclusion

CD44 is a transmembrane protein with a variety of functions depending on its ligand binding, co-receptor associations, and cytoskeletal interaction. The normal functions of LMM HA binding with CD44 coupled with CD44’s inherent interactions with secondary signaling complexes allow for wound healing, modulation of the immune system, and developmental angiogenesis. Irregular or prolonged CD44 and CD44–HA interaction, however, can lead to fibrosis, scarring, immunopathology, and tumor growth. The high expression of CD44 and high production of HA in tumor environments make them both prime targets for therapeutic intervention. The presence of CD44 at high concentrations in the skin and other tissues and the necessity of HA production for proper wound healing complicates potential clinical intervention strategies. Further understanding of the complexities of CD44 HA-dependent and independent signaling are required for developing highly specific targeted therapeutics that avoid the previously seen serious adverse reactions.

## Conflict of Interest Statement

The authors declare that the research was conducted in the absence of any commercial or financial relationships that could be construed as a potential conflict of interest.

## Abbreviations

CSC, cancer stem cell; EMT, epithelial–mesenchymal transition; ERM, ezrin–radixin–moesin; GAS, group A *Streptococcus*; HA, hyaluronic acid; HCV, hepatitis C virus; HGF, hepatocyte growth factor; HMM, high molecular mass; ILD, interstitial lung disease; IPF, idiopathic pulmonary fibrosis; LMM, low molecular mass; MMP, matrix metalloproteinase; milk-HA, HA derived from breast milk; RHAMM, receptor for hyaluronic acid-mediated motility; TLR, toll-like receptor.
